# Supplementary material for: Insights into the Peritumoural Brain Zone of Glioblastoma: CDK4 and EXT2 May Be Potential Drivers of Malignancy
Source: Int J Mol Sci. 2023 Feb 2;24(3):2835. doi: 10.3390/ijms24032835 (PMC9917451; doi:10.3390/ijms24032835)
Supplement: Supplementary file 1 [file ijms-24-02835-s001.zip › ijms-2101156-supplementary.pdf]

Supplementary Material

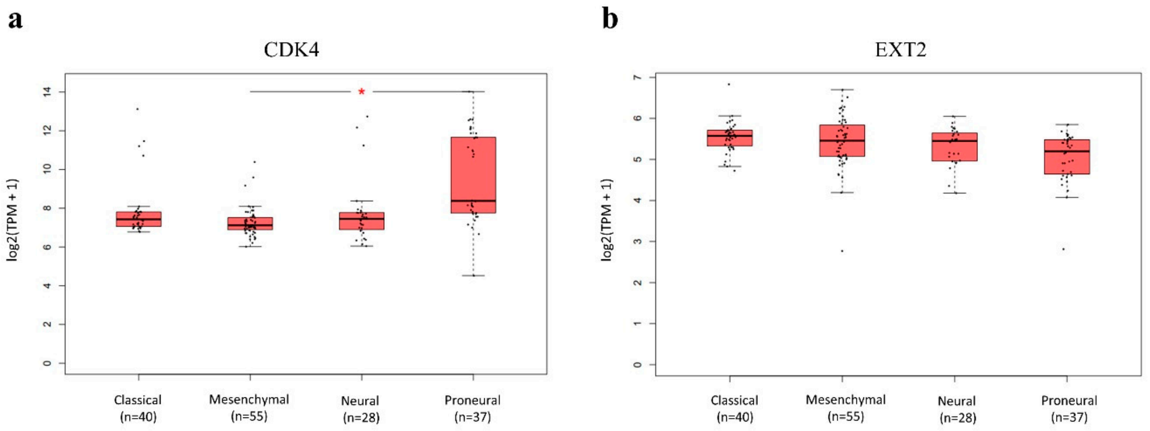

Figure S1. Differential *CDK4* (**a**) and *EXT2* (**b**) mRNA expression among glioblastoma molecular subtypes in the GEPIA2 database. TPM: Transcripts Per Kilobase of exon model per Million mapped reads. * $p < 0.05$.

a

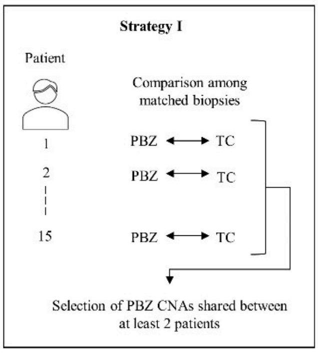

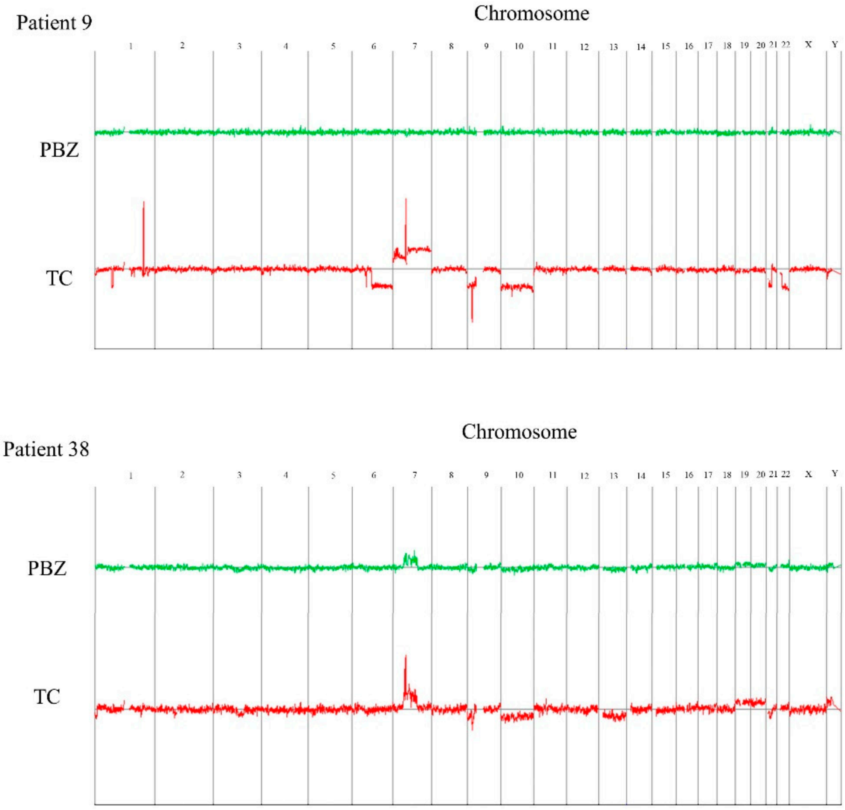

Example: comparison between PBZ and TC genomic profiles of patient 9. Identification of CN gain in 11p11.2 locus specifically in PBZ. Identification of the same unique CNA in the PBZ of patient 38. Selection of 11p11.2 gain as interesting CNA of the PBZ by strategy I.

b

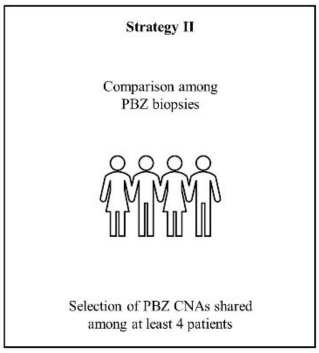

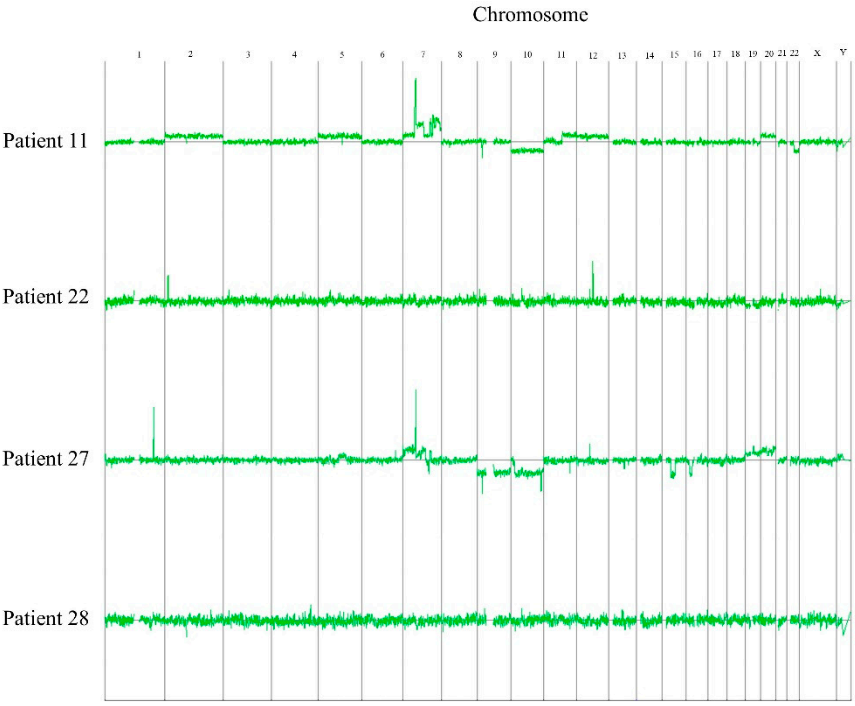

Example: comparison of PBZ genomic profiles of patient 11, 22, 27 and 28. Identification of CN gain in 7q21.12 locus shared among the patients. Selection of 7q21.12 gain as interesting CNA of the PBZ by strategy II.

Figure S2. Selection of CNAs. Summary and related examples of the strategies adopted in the selection of the copy number alterations (CNAs) of interest for the peritumoural brain zone (PBZ), identified by studying the genomic profiles of the tumour core (TC) biopsies and the related PBZ ones. (**a**) strategy I; (**b)** strategy II.

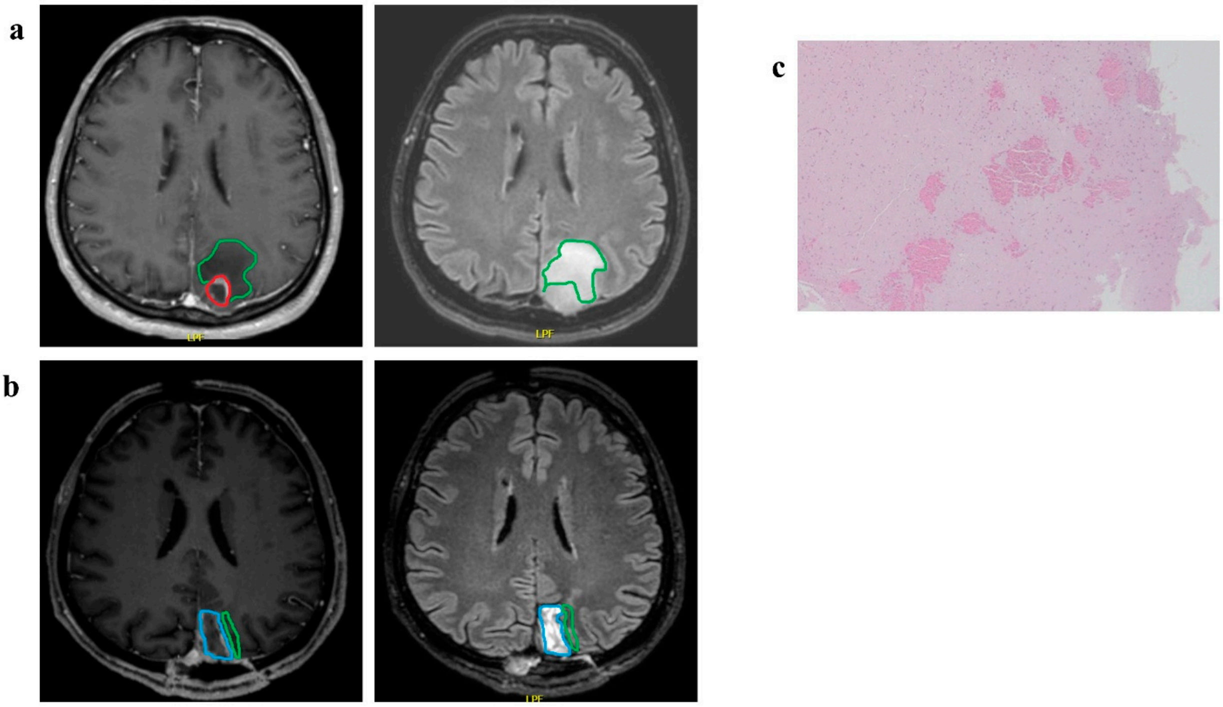

Figure S3. Peritumoral brain zone (PBZ) identification. (**a**) Pre-operative Magnetic Resonance Imaging (MRI) (T1 with gadolinium and FLAIR weighted images). In red, the tumour core (TC). In green, the PBZ. The sampling of the PBZ tissue was performed within the green area according to the intraoperative tumour features. (**b**) Post-operative MRI (T1 with gadolinium and FLAIR weighted images). In blue, the surgical cavity. In green the site for PBZ sampling. (**c**) Haematoxylin-eosin staining of the PBZ tissue sampled during the surgery is negative for tumour infiltration.
